# Enhanced H_2_ production from dehydrogenation of sodium borohydride over the ternary Co_0.97_Pt_0.03_/CeO_*x*_ nanocomposite grown on CGO catalytic support†

**DOI:** 10.1039/c9ra10742h

**Published:** 2020-10-16

**Authors:** Abhay Vijay Kotkondawar, Sadhana Rayalu

**Affiliations:** Environmental Materials Division, CSIR-National Environmental Engineering Research Institute Nehru Marg Nagpur-440020 Maharashtra (M.S.) India s_rayalu@neeri.res.in chemabhay.17@gmail.com

## Abstract

The development of low-cost materials for the 100% dehydrogenation of metal hydrides is highly essential to vitalize the chemical hydride-based hydrogen economy. In this context, the ternary Co–Ce–Pt nanocomposite immobilized on functionalized catalytic support CGO is synthesized by the one step chemical reduction approach and has been directly employed for the ethanolysis of sodium borohydride. The co-operative effect of CGO and the synergy between metallic nanoparticles is investigated to determine the highest rate of hydrogen (H_2_) production. The maximum hydrogen generation rate (HGR) of 41.53 L (min g_*M*_)^−1^ is achieved with the Co_0.97_Pt_0.03_/CeO_*x*_/CGO nanohybrid from the alkaline ethanolysis of sodium borohydride (SB). In addition, the resultant nanohybrid exhibited a relatively low activation energy of 21.42 kJ mol^−1^ for the ethanolysis of SB. This enhanced catalytic activity may be attributed to the intermetallic charge transport among metallic Pt, Co/Co_3_O_4_, and CeO_*x*_ counterparts. Moreover, the catalytic support CGO provides mesoporous functionalized surface and its intercalated GO layers promote charge transport. These results indicate that the resultant catalytic system described here for the dehydrogenation of SB can offer a portable and low-cost H_2_ supply for various fuel cell applications.

## Abstract 2

Synergistic interactions among Co/Co_3_O_4_, CeO_*x*_ and Pt counterparts, and the co-operative effect of CGO play a significant role in the complete dehydrogenation of sodium borohydride.

## Graphical abstract

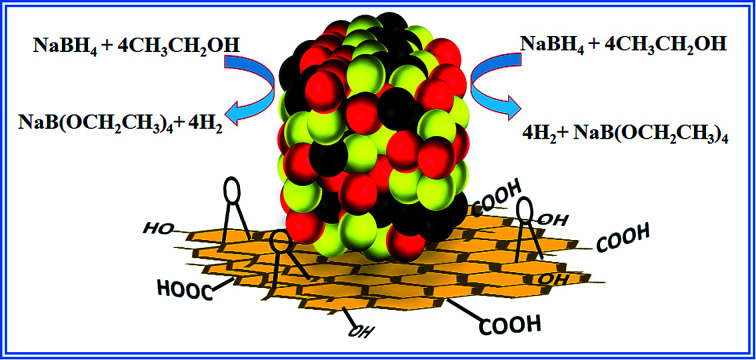

## 1. Introduction

“Hydrogen economy” is the most fascinating energy portfolio owing to its high energy density (142 MJ kg^−1^) and zero carbon emission, which can satisfy the current increasing demand for energy. However, its proficiency for niche applications is mainly restricted due to inadequate molecular hydrogen (H_2_) supply, its vital storage, and facile transport.^1^ Nevertheless, various H_2_ production routes are available but the dehydrogenation of chemical hydrides is the most attractive option to realize the high volume of H_2_ production.^2^ Among chemical hydrides, sodium borohydride (SB) is commended as the most promising candidate for H_2_ supply owing to its high metal hydrogen storage capacity (10.8 wt%), low operating temperature, and hydrogen purity.^3^ In general, H_2_ production from SB proceeds *via* catalyzed hydrolysis reactions, according to eqn (1).

1 NaBH_4_ + 2H_2_O → 4H_2_ + NaBO_2(aq)_

The hydrolysis byproduct, *i.e.*, NaBO_2_, exhibits very low solubility in an aqueous medium, which usually causes the plugging problem in the reactor and its excess accumulation can deteriorate the active catalytic sites. Therefore, primary alcohols are proposed as alternative solvotic agents.^4,5^ In particular, ethanol, with its low toxicity and production dependency on natural bio-resources, is an environmentally benign solvotic agent.^6,7^ Moreover, ethanolic dehydrogenation reactions (eqn (2)) can be operated at sub-zero temperatures and most importantly, their by-products are easy to convert back to their parental form.^8,9^

2 NaBH_4_ + 4CH_3_CH_2_OH → 4H_2_ + NaB(OC_2_H_5_)_4_

Along with the solvotic medium, the catalyst and catalytic support material show a significant influence on the reaction kinetics and degree of dehydrogenation. So far, various catalytic compositions comprised of non-noble metal nanoparticles, *i.e.*, Co,^10,11^ Fe,^12^ Ni,^13^ Cu,^14^ and noble metal nanoparticles, *i.e.*, Pt,^15,16^ Pd,^17,18^ Au,^19^ and Ru,^20–22^ have been extensively explored for the dehydrogenation of SB. Amongst them, cobalt nanoparticles have attracted more attention owing to their high affinity towards BH_4_^−^ ions and nanoscale magnetic behavior.^10^ Moreover, the incorporation of non-metals, *i.e.*, Co–B,^11^ Co–P–B,^23^ Co–Ni–P–B,^24^ Co–P–Pd,^25^ or/and doping of transition/inner transition metal, *i.e.*, Co–W–B,^26^ Co–Zn–B,^27^ Co–La–Zr–B,^28^ and Co–Ce–B,^6^ was found to accelerate hydrolysis/ethanolysis reactions either by controlling the growth of Co-NP or by increasing the synergistic interaction between the metal NPs. Compared to mono and bi-metallic counterparts, much better selectivity and high catalytic activity were demonstrated for the trimetallic composites, *i.e.*, Ni_45_Co_45_Au_10_ (ref. 29) and Ni_40_Fe_40_Pd_20_.^30^ In the trimetallic composite, multiple co-ordinations amongst metals provide new active sites (geometric effect) and their intermetallic charge transfer changes the individual electronic properties (electronic effect).^11,31,32^ These two effects significantly enhance the overall catalytic activity and both the effects are often operated synergistically. Therefore, to avail such a synergistic effect, we have synthesized the ternary Co_0.97_Pt_0.03_/CeO_*x*_ heterostructure by the one-step co-reduction approach. The CeO_*x*_ counterpart is deliberately incorporated into the structure owing to its variable oxidation state, which changes spontaneously on interaction with other metal ions.^33,34^ The interchangeable valence state usually promotes electron transport among metallic counterparts. Furthermore, the presence of Ce(iii) defects can develop extra negative charge around the oxide surface, which could enhance the co-ordination of metal nanoparticles to the oxide surface.^35–37^

It is observed that the tuning of metallic NPs with a catalyst support can further accelerate the catalytic performance by providing functionalized anchoring sites for the nanoparticles by maximizing the specific surface area and preventing them from agglomeration.^38^ Amongst the available catalyst support materials, the carbon family member's, namely, carbon nanodot (0D),^39,40^ carbon nanotube (1D),^18^ graphene oxide,^41^ and graphene (2D),^42^ have received immense attention owing to their chemical inertness, hydrophobic nature, thermal stability, low corrosivity, and the availability of an easily functional high surface area. On the other hand, amorphous carbon (carbon black), which is often considered as an impurity, is comparatively less studied as a catalyst or catalytic support material.^43^ Its amorphous phase, irregular morphology, and lacking surface functionality restricts its catalytic applicability. Therefore, to modify the amorphous carbon, herein, we have synthesized carbon-grafted graphene oxide (CGO) catalyst support materials by the extensive chemical oxidation of amorphous carbon and graphite powder.^44^ This oxidative treatment not only reduces the interface between the carbon and graphene oxide but also develops mesoporous channels, which provide active sites for metal NPs loading.

In the present work, we have developed novel functionalized catalytic support CGO by the exfoliation of amorphous carbon and graphite powder in acidic media. It was further employed as a catalyst support for the impregnation of the ternary Co–Ce–Pt nanocomposite by a one-step chemical reduction approach. The synergy of CGO and ternary Co–Ce–Pt nanocomposite has been tested for hydrogen production *via* alkaline ethanolysis of sodium borohydride at 300 K. The evaluation parameters were optimized with respect to the different metallic molar ratio of Co, Ce, and Pt, catalyst support, solvotic medium, and SB concentrations to determine the highest rate of H_2_ production. The details have been discussed as follows.

## 2. Experimental section

### 2.1. Chemicals

Sodium borohydride (98%), conc. sulfuric acid (98%), conc. phosphoric acid (35%), ethanol, and hexa-chloroplatinic acid [H_2_PtCl_6_·6H_2_O, 40% Pt] were purchased from Merck, India Limited. Cobalt nitrate(ii) hexahydrate [Co(NO_3_)_2_·6H_2_O, 99.99%] and cerium nitrate(iii) hexahydrate [Ce(NO_3_)_3_·6H_2_O, 99.99%] were procured from Sigma-Aldrich. Graphite powder was obtained from the nano-shell. The synthesis of carbon nanoparticles (CNP) has been described in our previous article.^45^ All the chemicals were used as obtained without further purification. Ordinary distilled water was used for synthesis and evaluation studies.

### 2.2. Synthesis of carbon-grafted graphene oxide (CGO)

The catalytic support CGO was synthesized by the chemical oxidation of carbon nanoparticles (CNP) and graphitic powder by modified Hummers' method with a slight modification in the purification step.^44^ In a typical synthesis, 2 g of CNP and 1 g of graphite powder were mixed mechanically and dispersed in (9 : 1) volume ratio of H_2_SO_4_ and H_3_PO_4_ in 1 L of flask. Then, 18 g of KMnO_4_ was slowly added to the acid mixture with continuous stirring in the temperature range of 40–45 °C. After continuous stirring for 30 min, the resultant mixture was refluxed for 12 h at 70 °C. On the completion of oxidative treatment, the reaction was quenched by the addition of 400 mL of ice water along with 5–6 drops of 30% H_2_O_2_. The resultant solution was allowed to settle overnight; the slurry was centrifuged to obtain the sedimented product. The resultant composite was repeatedly washed with acetone–water mixture until the pH was neutral. The composite was dried at 60 °C under vacuum.

### 2.3. Synthesis of Co_0.97_Pt_0.03_/CeO_*x*_/CGO nanohybrid

Typically, 30 mg of CGO was used as a support material for the anchoring metal nanoparticles. CGO was dispersed in 4 mL of ethanol solution and all the three-metal precursors, namely, Co(NO_3_)_2_·6H_2_O, Ce(NO_3_)_3_·6H_2_O, and H_2_PtCl_6_·6H_2_O were added to the same suspension at the concentration of 0.32 mM, 0.028 mM, and 0.0019 mM, respectively. The mixture was sonicated for 30 min to form a homogenous dispersion. Then, 13.21 mM alkaline (12 wt% NaOH) NaBH_4_ solution was added to the above mixture with vigorous stirring. The alkaline solvotic medium suppresses the self-hydrolysis of NaBH_4_ in ethanol medium. Once the formation of H_2_ bubble ceased, the black suspension of the Co_0.97_Pt_0.03_/CeO_*x*_/CGO nanohybrid was obtained, which was used as a catalyst for further dehydrogenation reactions. All the experimental evaluation was carried out in a 125 mL glass vial sealed with a rubber septum connected with an inverted measuring cylinder filled with water. The ethanolysis of SB was optimized by varying the molar concentration of Co^2+^, Ce^3+^, and Pt^4+^ under constant experimental conditions.

### 2.4. Catalytic ethanolysis, reusability, and recyclability of catalyst

The catalytic potential of the as-synthesized nanohybrid, *i.e.*, Co_0.97_Pt_0.03_/CeO_*x*_/CGO, Co_0.97_Pt_0.03_/CeO_*x*_, and Co/CeO_*x*_/CGO, Co/CGO towards the ethanolysis of SB and the amount of H_2_ generated was evaluated by the water displacement method. Typically, a 125 mL cylindrical glass vial with a catalyst3
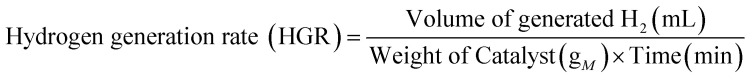
*M* = weight of Co, Ce and Pt components in nanocomposite suspension is placed in a water bath at 27 ± 2 °C and connected to a water-filled inverted measuring cylinder. The catalytic activity of the nanohybrid for the ethanolysis of SB was estimated in terms of HGR, as calculated by eqn (3). The reusability test of the nanohybrid has been performed by consecutively injecting 13.21 mM of alkaline SB after each successive cycle in the same reaction mixture under similar experimental conditions. The recyclability experiment was performed by reusing the catalyst after washing before the next run.

### 2.5. Catalyst characterization

Powder X-ray diffraction pattern (XRD) of the synthesized nanohybrid has been analyzed with a Rigaku Miniflex-II diffractometer using Cu Kα_1_ radiation at 30 kV and 15 mA with a monochromator. The diffraction pattern was recorded between 6 and 80° with a scanning speed of 3° min^−1^. The X-ray diffractograms were compared with a standard JCPDS card. The surface morphology of the nanohybrid and the arrangement of NPs at the atomic scale was examined using a transmission electron microscope (TEM) (FEI, Tecnai T-20) operated at 200 kV. A high angle annular dark field (HADDF) detector was used for the scanning transmission electron microscopic (STEM) experiment. The FT-IR of the prepared catalysts was recorded with the Bruker Model – 152 Vertex-70 spectrophotometer using the KBr pellet technique. Raman measurements were obtained from a Renishaw Raman spectrophotometer. The Brunauer–Emmett–Teller (BET) specific surface area and Barrett–Joyner–Halenda (BJH) pore size distribution was evaluated using a Quantachrome Nova Touch NT1.1 surface area and a pore size analyzer, respectively. The samples were degassed at 200 °C for 2 h to eliminate the physically adsorbed water and the atmospheric impurities prior to adsorption–desorption isotherm measurements. The samples for electron microscopy were prepared by dispersing the powder in ethanol and coating a very dilute suspension on carbon-coated Cu grids. Scanning Electron Microscopy (SEM) images and local elemental composition were determined using an FEI QUANTA 200 microscope and coupled with an energy dispersive X-ray analysis (EDX). The electronic structure of the synthesized bimetallic nanohybrid was investigated using high-resolution X-ray photoelectron spectroscopy (XPS). The measurement was performed using a VG Microtech Multilab ESCA 3000 spectrometer with a non-monochromatized Mg Kα X-ray radiation. All the binding energies (BEs) were normalized using the adventitious carbon (C 1s = 284.4 eV) as a reference. For further analysis, the peaks were deconvoluted by Voigt function.

## 3. Results and discussion

### 3.1. Synthesis and characterization of the sample

**Fig. 1:**
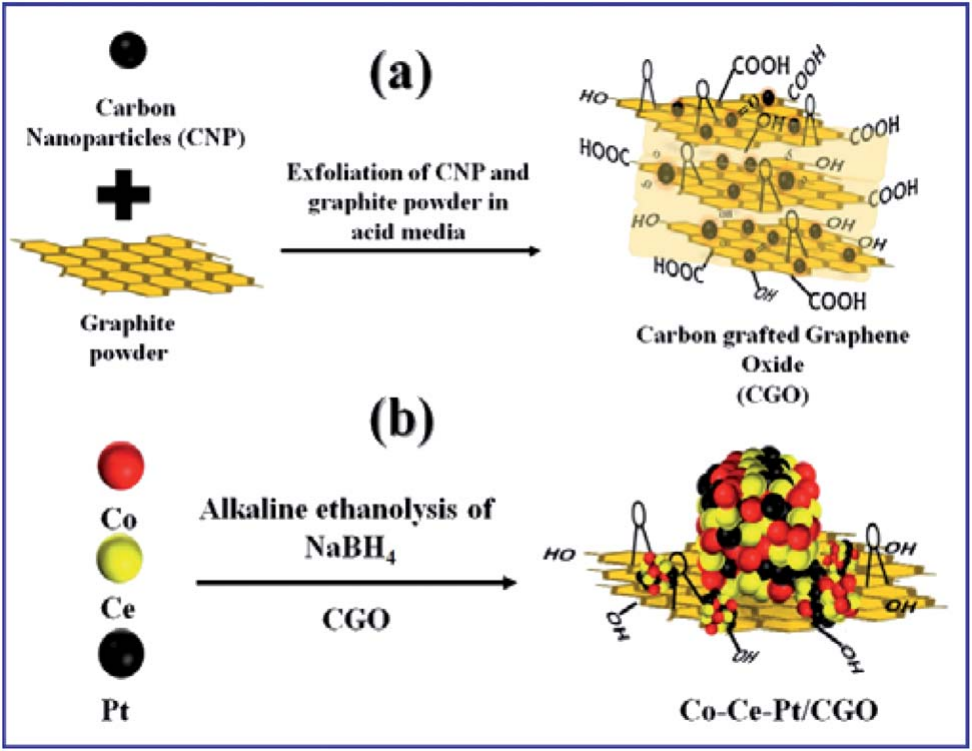
Pictorial representation for the formation of (a) CGO and (b) Co_0.97_Pt_0.03_/CeO_*x*_/CGO.

In a typical synthesis, the ternary nanocomposite with different metallic molar ratio of Co, Ce, and Pt was grown on the CGO support by the facile chemical reduction approach using NaBH_4_ as a reducing agent in an alkaline (12 wt% NaOH) ethanol–water medium at 300 K. The synthesis of CGO and ternary CoPt/CeO_*x*_/CGO nanocomposite is represented in Fig. 1. Among the salts in the mixture, the Ce(iii) ions are more susceptible to hydroxylation while the Co(ii) and Pt(iv) ions can be deposited in their metallic state. On the addition of alkaline NaBH_4_ solution, the generated black colored solution represented that the Pt(iv) and Co(ii) ions could get reduced to their metallic state. The released heat during the course of reduction and dissolved oxygen of the solution could convert the Ce(iii) hydroxylates into the amorphous CeO_*x*_ oxide phase. In an alkaline environment, it may possible that some of the metallic cobalt may transform into the hydroxylate and with the aid of generated heat and could form the Co_*x*_O_*y*_ oxide.^25,46^ The instantaneously generated stream of H_2_ largely influences the crystallinity of the reduced metallic nanoparticles.

In addition, it may facilitate the reduction of oxidized Co_*x*_O_*y*_ to its metallic state. Therefore, it can be inferred that the *in situ* generated metallic Co, Pt, and amorphous CeO_*x*_ nanoparticles served as the seeds for the successive growth of the ternary CoPt/CeO_*x*_ nanocomposite on the CGO support. The resultant CoPt/CeO_*x*_/CGO nanohybrid has been tested for alkaline ethanolysis of SB at 300 K. Among all the tested metallic variations, the Co_0.97_Pt_0.03_/CeO_*x*_/CGO nanohybrid have shown the highest catalytic activity for dehydrogenation; therefore, it was used as a reference catalyst for instrumental characterization. The synthetic recipe and chemical composition of the Co, Ce, and Pt elements (%wt) in the Co_0.97_Pt_0.03_/CeO_*x*_/CGO nanocomposite are presented in Table 1.

**Table 1:** Reaction recipe and the characterization details of the Co_0.97_Pt_0.03_/CeO_*x*_/CGO nanohybrid and the details of hydrogen production rate from the ethanolysis of SB at 300 K

Catalyst	[Co] mM	[Ce] mM	[Pt] mM	%wt Co	%wt Ce	%wt Pt	Ethanolysis of sodium borohydride	
							Average completion time (min)	HGR [L(H_2_) (min g_*M*_)^−1^]
**Co**_**0.97**_ **Pt**_**0.03**_ **/CeO**_***x***_ **/CGO**	0.32	0.028	1.9 × 10^−3^	56.2	12.75	1.05	1.31	41.53
**Co**_**0.97**_ **Pt**_**0.03**_ **/CeO**_***x***_	0.32	0.028	1.9 × 10^−3^	52.5	16.45	1.05	2.87	19.02

**Fig. 2:**
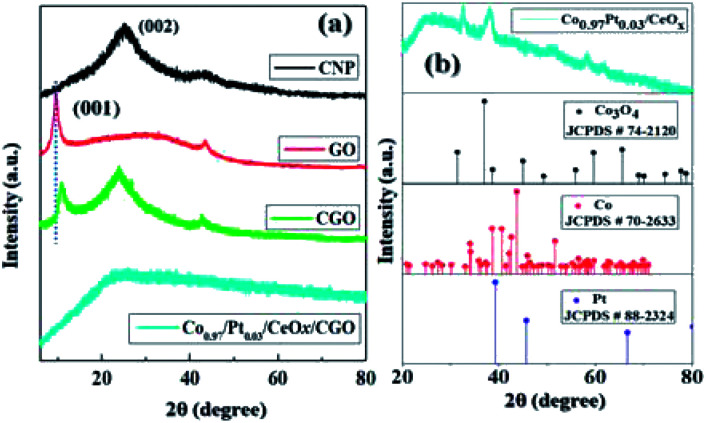
The XRD pattern of (a) CNP, GO, CGO, and (b) Co_0.97_Pt_0.03_/CeO_*x*_ [the vertical dotted line represents the shift in the peak position (001) between GO and CGO].

The powder XRD pattern of CNP, GO, CGO, and Co_0.97_Pt_0.03_/CeO_*x*_ are illustrated in Fig. 2(a) and (b). It was observed that the synthesized CGO exhibited both the characteristics of GO and CNP. The intense peak at about 10.74° represents the existence of graphene oxide, which originates from the reflection of the (001) plane, while a broad peak at 24.8° belongs to the diffraction pattern of amorphous carbon.^39,47^ Using Bragg's law, the interlayer spacing of GO and CGO for the (001) plane was calculated as 10.19 Å and 9.47 Å, respectively. Compared to GO, the observed slight shift in the peak position of CGO and the decreased *d*_(001)_ value indicates the co-intercalation of amorphous carbon, nitrate, and sulfate in the graphitic layer during extensive chemical oxidation.^48^ For Co_0.97_Pt_0.03_/CeO_*x*_/CGO, a very weak XRD signal was observed, which indicates the formation of ultrafine nanoparticles occurring during the one-step reduction process or their embedment under CGO layers. XRD measurement without the support, *i.e.*, Co_0.97_Pt_0.03_/CeO_*x*_, showed the dominant diffraction pattern of Co_3_O_4_ [JCPDS # 74-2120],^49^ while the presence of metallic Co, Pt, and CeO_*x*_ counterparts remains undetected, probably due to the low resolution of the instrument. Further, the surface functional groups of CGO and its degree of graphitization were evaluated using Fourier transform infrared (FT-IR) and Raman spectroscopic technique, respectively.

**Fig. 3:**
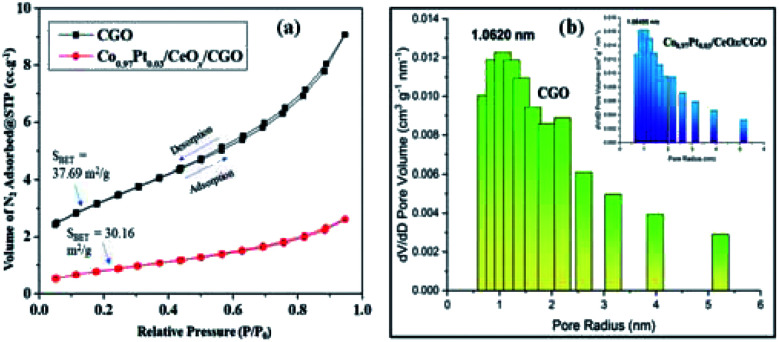
(a) N_2_ adsorption–desorption isotherm of CGO and Co_0.97_Pt_0.03_/CeO_*x*_/CGO. (b) Pore size distribution of CGO and Co_0.97_Pt_0.03_/CeO_*x*_/CGO (inset).

The relative FT-IR spectrum of CNP and CGO is described in Fig. S1.† The IR stretching frequencies at 1720 cm^−1^, 1590 cm^−1^, 1250 cm^−1^, and 870 cm^−1^ represent the symmetric vibration of C=O, C=C, C–O, and C–OH bonds, respectively.^44,47^ These obtained functionalities may allow the homogenous distribution of metallic nanoparticles. The Raman spectrum of CNP, CGO, and Co_0.97_Pt_0.03_/CeO_*x*_/CGO are shown in Fig. S2.† In CGO, the distinct D and G band situated at 1346 cm^−1^ and 1584 cm^−1^ confirmed the formation of the graphene oxide phase.^41,42^ The increased intensity ratio of the D and G band in Co_0.97_Pt_0.03_/CeO_*x*_/CGO (*I*_D_/*I*_G_ = 1.19) compared to CGO (*I*_D_/*I*_G_ = 1.06) could suggest the formation of more sp^2^ hybridized domain on NaBH_4_ reduction.^50^ The alliance of the increased graphene domain may promote the intermetallic charge transport among the metallic counterparts.^27,47^ The specific surface area and pore size of the as-prepared catalyst was studied by N_2_-adsorption analysis. The Brunauer–Emmett–Teller (BET) specific surface areas of CGO and Co_0.97_Pt_0.03_/CeO_*x*_/CGO were 37.12 and 30.16 m^2^ g^−1^, respectively. Compared to the support, the lower surface area of the composite is due to the blocking of the CGO pores by metal NPs during preparation. As shown in Fig. 3(a), the N_2_ adsorption–desorption isotherm of CGO and Co_0.97_Pt_0.03_/CeO_*x*_/CGO reflected type-IV isotherms with a hysteresis loop observed within the relative pressure range of 0.7–0.9. The pore size distribution in CGO and Co_0.97_Pt_0.03_/CeO_*x*_/CGO [Fig. 3(b)] was analyzed with the Barrett–Joyner–Halenda (BJH) technique, which shows the maxima in the mesoporous region with an average pore size of 1.06, which corroborates the mesoporous nature of CGO and the trimetallic nanohybrid.

**Fig. 4:**
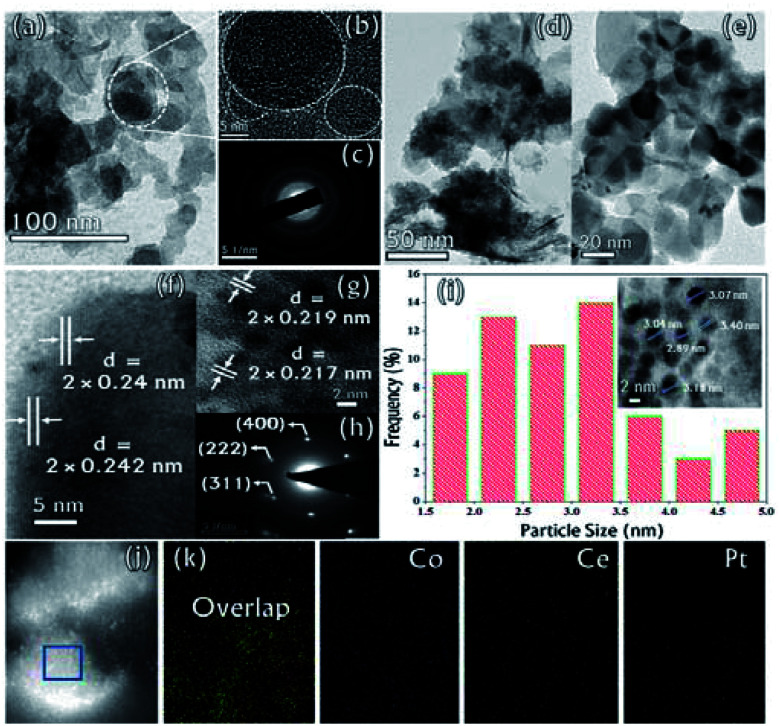
(a) Low and (b) high magnification TEM image of CGO, (c) SAED pattern of CGO, (d) low and (e) high magnification TEM images of the Co_0.97_Pt_0.03_/CeO_*x*_/CGO nanohybrid, (f) and (g) the HR-TEM image of Co_0.97_Pt_0.03_/CeO_*x*_/CGO, (h) the SAED pattern of Co_0.97_Pt_0.03_/CeO_*x*_/CGO, (i) the particle size histogram and the inset TEM image of Co_0.97_Pt_0.03_/CeO_*x*_/CGO, (j) the HAADF-STEM image and the corresponding (k) elemental mapping for Co_0.97_Pt_0.03_/CeO_*x*_/CGO.

**Fig. 5:**
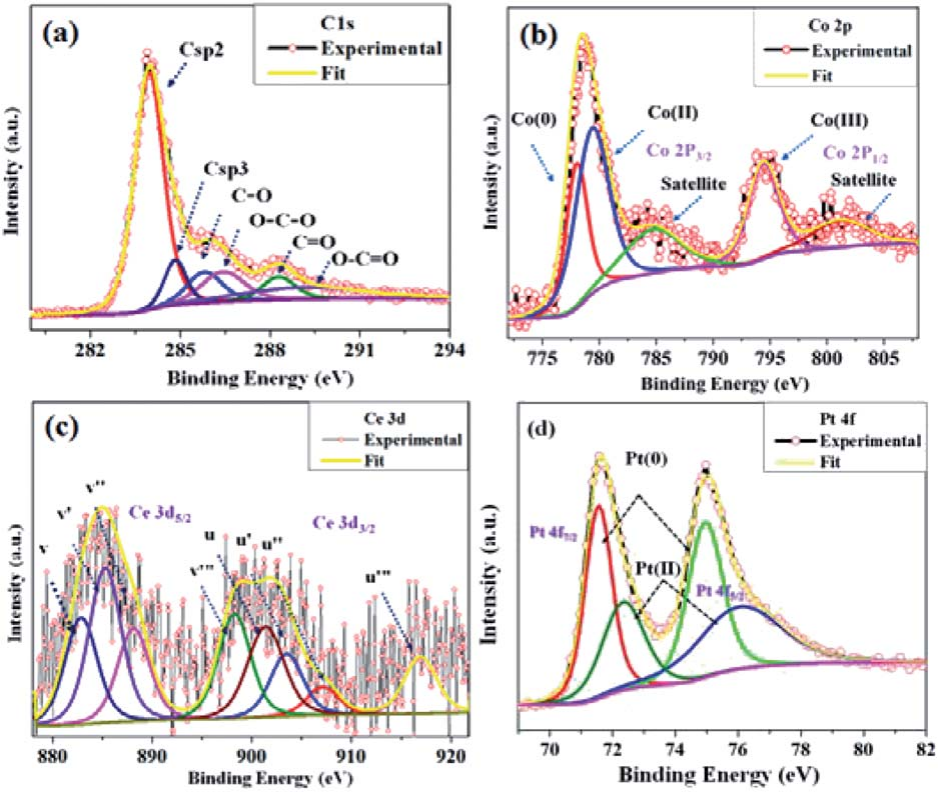
High-resolution XPS spectrum of (a) C 1s from CGO and (b) Co 2p, (c) Ce 3d, and (d) Pt 4f from the Co_0.97_Pt_0.03_/CeO_*x*_/CGO nanohybrid.

The microstructure of the synthesized CGO was examined by Transmission Electron Microscopy (TEM). The low-resolution TEM image [Fig. 4(a)] shows that the CGO particles are fused together in a chain-type structure with irregularity at the edges. These irregularities may appear due to extensive chemical oxidation. The HR-TEM image shown in Fig. 4(b) represents the distorted onion-type structure of CGO and also shows the presence of mesoporous channel in the carbon network. The crystallinity of CGO can be clearly seen from the SAED pattern [Fig. 4(c)]; the starting diffuse ring may correspond to amorphous carbon while the last ring represents the spacing between the graphitic planes.^51^ Therefore, it can be inferred that CGO exhibits both the characteristics of amorphous carbon and crystalline graphene oxide. Along with this, the preparative exfoliation technique in acidic media facilitates the creation of mesopores, which can enhance the overall surface accessibility. These observations were in good accordance with XRD and N_2_-adsorption–desorption analysis. On the other hand, the TEM image of the Co_0.97_Pt_0.03_/CeO_*x*_/CGO nanocomposite shown in Fig. 4(d) indicates the fine distribution of metallic nanoparticles on the surface of CGO, which was generated during the ethanolysis of SB. On close resolution [Fig. 4(e)], it was observed that most of the metallic nanoparticles were embedded in the CGO matrix. The HR-TEM image shown in Fig. 4(f and g) revealed the existence of cobalt and platinum. The two lattice fringes with the *d* spacing of 0.242 nm (JCPDS #74-2120)^49^ and 0.219 nm (JCPDS #88-2343)^52^ can be assigned to Co_3_O_4_ (311) and the metallic Pt NPs, respectively. Compared to the JCPDS database, the observed downshift value in the *d*-spacing of Pt nanoparticles could suggest the dominant interaction among the Pt, Co, and CeO_*x*_ counterparts.^53^ The corresponding SAED pattern [Fig. 4(h)] showed that Co_*x*_O_*y*_ has a higher degree of crystallinity compared to other metallic components. The *d*-spacing value of 0.240 nm calculated from the SAED pattern confirmed the presence of crystalline Co_3_O_4_ (311). Regardless of the co-existence of cobalt and platinum, the presence of cerium was unable to be detected by XRD and TEM analysis. The relative histogram of the particle size *versus* % distribution of metallic NPs is described in Fig. 4(i) and the inset TEM image showed that the as-synthesized metallic NPs have an extremely small size of 2–3 nm. The high-angle annular dark-field scanning transmission electron microscopic (HADDF-STEM) and the elemental mapping images for the Co_0.97_Pt_0.03_/CeO_*x*_/CGO nanohybrid are shown in Fig. 4(j) and (k), respectively. It represented the dispersion of Co, Ce, and Pt elements on the surface of CGO with a higher abundance of Co element. The corresponding energy dispersive X-ray (EDX) spectrum further confirmed the existence of Co, Ce, Pt, C, and O elements in the Co_0.97_Pt_0.03_/CeO_*x*_/CGO nanohybrid (Fig. S3†). The surface functionality and oxidation state of the metallic nanoparticles were investigated by X-ray photoelectron spectroscopy. In Fig. 5(a), the high-resolution C 1s spectrum is deconvoluted into six different components labeled from C-1 to C-6. The peaks centered at 283.96 eV and 285.4 eV could be associated with the sp^2^ and sp^3^ hybridized carbon.^54,55^ Nearly 54% of higher sp^2^ hybridized carbon was obtained in CGO. In addition to this, the surface oxygen functionalities such as C–O (285.8 eV), O–C–O (286.4 eV), and C=O (288.28 eV) were found on the surface of CGO.^54,56^ The higher BE of 289.06 eV can be assigned to O–C=O or aromatic C=C π-functional groups (π–π* interaction).^57^ The XPS investigation of atomic oxygen (O 1s) also confirmed the formation of oxygen functionality on the surface of CGO (Fig. S4†). The peaks situated at 531.01 eV and 532.40 eV could be assigned to C–O and C=O functional groups, respectively.^57^ The summary of functional groups obtained on the surface of CGO is represented in Table S1.† For the Co_0.97_Pt_0.03_/CeO_*x*_/CGO nanohybrid, the survey spectrum indicated the unambiguous presence of Co, Ce, and Pt elements (Fig. S5†).

The deconvoluted Co 2p XPS spectra shown in Fig. 5(b) revealed that the elemental cobalt exists in Co(0), Co(ii), and Co(iii) valence states with a ratio of 25.5%, 27.1%, and 47.4%, respectively. The peaks with B.E. of 778.08 eV (Co 2p_3/2_), 780.13 eV (Co 2p_3/2_), and 795.2 eV (Co 2p_1/2_) could be attributed to metallic cobalt (Co^0^), Co_2_O_3_ (Co^3+^), and CoO (Co^2+^) phases, respectively. The two satellite peaks at 785.6 eV (Co 2p_3/2_) and 803.96 eV (Co 2p_1/2_) further confirmed the formation of cobalt oxides (*e.g.*, Co_2_O_3_, CoO, and/or Co_2_O_3_).^58,59^ However, the binding energies for Co^3+^ and Co^2+^ were found to be well-matched with the reported value of Co^2+^ and Co^3+^ cation in the Co_3_O_4_ crystal.^59^ Therefore, it can be inferred that cobalt exists in the mixed oxide state (Co^3+^/Co^2+^), similar to the Co_3_O_4_ crystal. The core level XPS spectra of Ce 3d is represented in Fig. 5(c); on deconvolution, nearly eight peaks were observed, which was denoted in multiplets of (*u*) and (*v*). The peaks labeled as *u* (901.39 eV), *u*′′ (907.09 eV), and *u*′′′ (916.93 eV) were observed due to the ionization of the Ce^4+^ (3d_3/2_) energy level, while the peaks labelled with *v* (882.84 eV), *v*′′ (888.11 eV), and *v*′′′ (898.24 eV) represented the Ce^4+^ (3d_5/2_) ionization.^60,61^ The peaks at *u*′ (903.48 eV) and *v*′ (885.26 eV) could be attributed to the Ce^3+^ oxidation state with the energy level of 3d_3/2_ and 3d_5/2_, respectively. The probable percentage of the Ce^3+^ and Ce^4+^ oxidation states is calculated by comparing the area under each curve and it was observed that nearly 75–80% of Ce (3d) exists in the Ce^4+^ oxidation state and the remaining 25–20% portion occupied by the Ce^3+^ state. It was well explained that the presence of Ce^3+^ can boost the overall catalytic performance by exchanging the lattice oxygen among vicinal cobalt ions, which can promote cobalt ions to a higher valence state.^62,63^ This may enhance the rate of dehydrogenation since Co(iii) ions can offer more active sites for the dehydrogenation of SB molecules. Lastly, the valence state of platinum was estimated by the deconvolution of the core level XPS spectra of Pt 4f shown in Fig. 5(d). The pair of doublets at 71.72 eV, 74.97 eV (Pt 4f_7/2_) and 72.42 eV, 75.89 eV (Pt 4f _5/2_) could be assigned to the Pt(0) and Pt(ii) oxidation state, respectively.^15,64^ On comparing the relative peak areas, it was found that Pt(0) has more abundance than the Pt(ii) component. The observed positive shift in the BE of Pt(0) may be attributed to the chemical interaction between Pt and CGO, cobalt, or cerium ions.^53,64^ Thus, from the above, it can be inferred that platinum majorly exists in the metallic form while cobalt and cerium are available in their mixed oxide state. The deconvolution summary of the Co_0.97_Pt_0.03_/CeO_*x*_/CGO nanohybrid is represented in Table S2.†

### 3.2. Catalytic ethanolysis of sodium borohydride

**Fig. 6:**
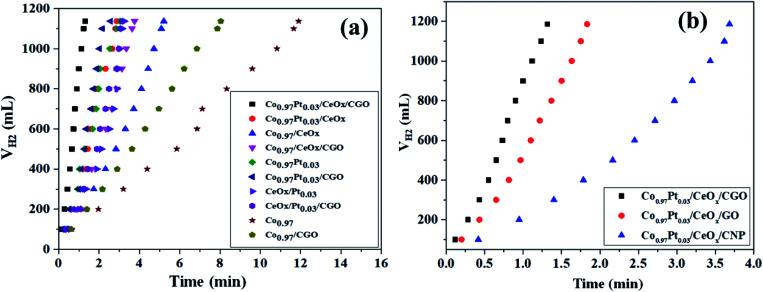
(a) Hydrogen generation from alkaline ethanolysis of SB (13.21 mM) catalyzed by Co_0.97_Pt_0.03_/CeO_*x*_/CGO, Co_0.97_Pt_0.03_/CeO_*x*_, Co_0.97_/CeO_*x*_, Co_0.97_/CeO_*x*_/CGO, Co_0.97_Pt_0.03_, Co_0.97_Pt_0.03_/CGO, CeO_*x*_/Pt_0.03_, CeO_*x*_/Pt_0.03_/CGO, free Co, and Co/CGO at 300 K. (b) Effect of the catalytic support S = CGO, GO, and CNP on catalytic activity of the Co_0.97_Pt_0.03_/CeO_*x*_/S nanohybrid for the alkaline ethanolysis of SB (13.21 mM).

The catalytic potential of the as-synthesized Co_0.97_Pt_0.03_/CeO_*x*_/CGO nanohybrid has been evaluated for the ethanolysis of SB under alkaline conditions (12 wt% NaOH) at 300 K. The series of nanocomposites with catalytic support (*i.e.*, Co_0.97_/CGO, Co_0.97_/CeO_*x*_/CGO, Co_0.97_Pt_0.03_/CGO, CeO_*x*_/Pt_0.03_/CGO, and Co_0.97_Pt_0.03_/CeO_*x*_/CGO) and without catalytic support (*i.e.*, Co, Co_0.97_/CeO_*x*_, Co_0.97_Pt_0.03_, CeO_*x*_/Pt_0.03_, and Co_0.97_Pt_0.03_/CeO_*x*_) have been extensively screened for the ethanolysis of SB to determine the catalytic dehydrogenation potential of each constituent ions. The evaluation profile for each combination is shown in Fig. 6(a). Nevertheless, all the metallic compositions have shown promising dehydrogenation potentials but the maximum hydrogen generation rate (HGR) of 41.53 L (min g_*M*_)^−1^ was obtained with the Co_0.97_Pt_0.03_/CeO_*x*_/CGO nanohybrid (Fig. S6†). This observed enhancement can be explained by Sabatier's principle,^65^ according to which the adsorption of BH_4_^−^ on the catalytic surface should not be too strong or too weak but should be at an appropriate proportion so that the maximum dehydrogenation reaction is facilitated on the active surface. On the Co_0.97_Pt_0.03_/CeO_*x*_/CGO nanohybrid, BH_4_^−^ ions can easily adsorb on the catalytic surface by interacting with the vacant d-orbital of the metal ions; in addition, the catalytic support CGO provides mesoporous channels for the lodging of NPs so that maximum sites are available for dehydrogenation reaction. Hence, a higher catalytic activity was observed with the ternary mixture compared to other mono and bimetallic compositions with and without the catalyst support. The significant role of CGO was further examined by evaluating the Co_0.97_Pt_0.03_/CeO_*x*_/CNP and Co_0.97_Pt_0.03_/CeO_*x*_/GO nanohybrids (*i.e.*, replacing CGO with CNP and GO) for the ethanolysis of SB; the activity profile is shown in Fig. 6(b). It was observed that 100% dehydrogenation has been obtained in all three cases but the highest HGR of 41.53 L (min g_*M*_)^−1^ was recorded for the Co_0.97_Pt_0.03_/CeO_*x*_/CGO nanohybrid (Fig. S7†).

This enhanced catalytic activity may be attributed to the (i) surface functionality present on the CGO, (ii) the availability of mesopores with ∼6 nm size in the carbon network and the high surface area of CGO (37.12 m^2^ g^−1^) compared to CNP that can facilitate the homogenous dispersion of the metallic nanoparticles, and (iii) the high stability of the CGO structure owing to the intercalated graphene oxide layers, which alleviates its agglomeration during the dehydrogenation reactions. In addition to the catalytic support, the solvotic medium has shown a significant influence on the hydrogen production [Fig. S8†]. Compared to the hydrolysis [21.39 L (min g_*M*_)^−1^], nearly two times higher hydrogen generation was observed with ethanol as a solvotic medium. This inherent acceleration may be ascribed to the better dispersibility of the Co_0.97_Pt_0.03_/CeO_*x*_/CGO nanohybrid in ethanol medium and the formation of a non-sticky by-product, *i.e.*, NaB(OCH_2_CH_3_)_4_, which reduces the plugging problem in the reactor.

**Fig. 7:**
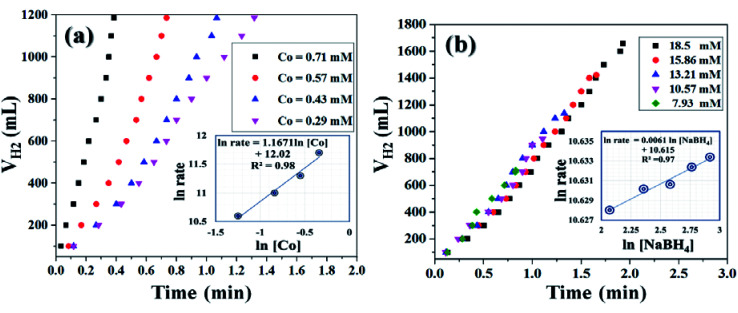
Hydrogen generation from the alkaline ethanolysis of SB (13.21 mM) catalyzed by Co_0.97_Pt_0.03_/CeO_*x*_/CGO with (a) different cobalt concentrations ranging from 0.71 mM to 0.29 mM and (b) different NaBH_4_ concentrations ranging from 7.93 mM to 18.5 mM at 300 K. The inset of each figure shows the logarithmic plot between the rate of H_2_ generation *versus* (a) cobalt concentration and (b) NaBH_4_ concentration.

**Fig. 8:**
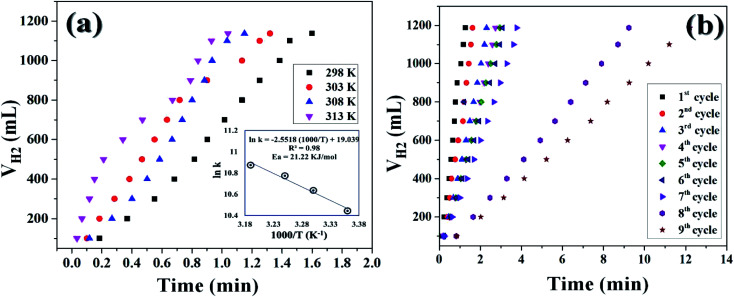
(a) Hydrogen generation from the alkaline ethanolysis of SB (13.21 mM) catalyzed by Co_0.97_Pt_0.03_/CeO_*x*_/CGO in the temperature range from 300 K to 313 K. The inset of the figure shows the logarithmic plot between the rate constant *versus* the inverse of temperature (K). (b) The reusability test of Co_0.97_Pt_0.03_/CeO_*x*_/CGO for hydrogen generation from the ethanolysis of SB.

It was already well investigated that the alkaline pH of the reaction medium restricts the self-hydrolysis of SB. The trend of H_2_ generation and the respective HGR from the ethanolysis of SB at different concentrations of NaOH is represented in Fig. S9 and S10,† respectively. It was observed that with the increase in NaOH concentration, the rate of hydrogen production increased linearly. However, at higher concentrations, *i.e.*, 12 wt% and 15 wt%, the increment is nearly same, which may be due to the inhibiting effect of hydroxide ions.^27^ By virtue of this, we have carried all the dehydrogenation reactions at an NaOH concentration of 12 wt% and the reaction temperature was maintained at 300 K. Thus, from the above evaluation studies, it can be inferred that Co_0.97_Pt_0.03_/CeO_*x*_/CGO is the most active nanohybrid for the ethanolysis of SB under alkaline conditions at 300 K. Further, the catalytic performance of the nanohybrid and the kinetics of ethanolysis have been optimized with respect to the concentration of cobalt, the concentration of NaBH_4_, and the reaction temperatures in similar reaction conditions. From Fig. 7(a), it can be seen that the rate of dehydrogenation is proportionality increased with the increase in the cobalt concentration from 0.29 mM to 0.71 mM. The highest hydrogen generation of 142.76 L (min g_*M*_)^−1^ was obtained for 0.71 mM of cobalt concentration; the respective trend of HGR with respect to cobalt concentration was depicted in Fig. S11.† The reaction order for the ethanolysis of SB at varying [Co] concentrations was determined by plotting the natural logarithmic plot of Co concentration against hydrogen generation [the inset in Fig. 7(a)]. The obtained well-fitted straight line with a slope value of 1.16 indicates that the reaction rate of ethanolysis is first order with respect to cobalt concentration. Similarly, the effect of NaBH_4_ concentration on the ethanolysis of SB was studied by varying the initial concentration from 7.93 mM to 18.50 mM under constant reaction conditions, as shown in Fig. 7(b). A constant linear increment in H_2_ evolution was observed and the obtained slope value of 6 × 10^−2^ [the inset in Fig. 7(b)] inferred that the ethanolysis of SB follows zero-order kinetics within the given range of NaBH_4_ concentration.^66^ Furthermore, the effect of temperature on the ethanolysis of SB was investigated by evaluating the nanohybrid at different temperature ranges between 298 K to 313 K. It is quite obvious that with the increase in reaction temperature, the rate of H_2_ generation is increased due to the enhancement in catalytic activity. The highest HGR of 52.81 L (min g_*M*_)^−1^ was obtained at 313 K. The plot of reaction temperature (K) *versus* the volume of H_2_ (mL) is shown in Fig. 8(a) and the change in the value of HG against temperature (K) is depicted in Fig. S12.† The apparent activation energy of the Co_0.97_Pt_0.03_/CeO_*x*_/CGO nanohybrid was calculated from the following Arrhenius equation, ln(*k*) = ln(*A*) − (*E*_a_/*RT*)where, *k* = reaction rate constant [L (min g)^−1^], *A* = pre-exponential factor, *E*_a_ = activation energy, and *T* = reaction temperature (K).

The apparent activation energy was calculated by multiplying the slope value of the Arrhenius plot [*i.e.*, the plot between the inverse of reaction temperature, (1000/*T* (K^−1^)), with the natural logarithm of the temperature-dependent rate constant (ln(*k*)), as shown in the inset of Fig. 8(a)] and the universal gas constant (*R*). The apparent activation energy (*E*_a_) for Co_0.97_Pt_0.03_/CeO_*x*_/CGO was found to be 21.22 kJ mol^−1^, which is comparatively lower than many popular cobalt-based composites (Table 2).

**Table 2:** Comparison of the catalytic activity and activation energy of various catalysts for the dehydrogenation of sodium borohydride (SB)

Sr. no.	Catalyst	HGR L (g_*M*_ min)^−1^	Activation energy (kJ mol^−1^)	Reference
**1.**	**CND**	1.066	22.01	39
**2.**	**Co**_**50**_ **B**_**100**_	2.407	—	11
**3.**	**Co@3DGO**	4.394	37.42	41
**4.**	**TiO**_**2**_ **-supported Co–Ce–B**	4.506	29.51	6
**5.**	**Ni**_**40**_ **Fe**_**40**_ **Pd**_**20**_	3.343	—	30
**6.**	**Ru-SZ**	10	76	22
**7.**	**Porous Co–P–Pd**	4.21	—	25
**8.**	**Ru–Co–PEDOT/PSS**	40.1	37.42	21
**9.**	**Pt/CeO**_**2**_ **–Co**_**7**_ **Ni**_**2**_ **O**_***x***_	7.834	47.4	35
**10.**	**Co**_**0.97**_ **Pt**_**0.03**_ **/CeO**_***x***_ **/CGO**	**41.53**	**21.22**	**This work**

**Fig. 9:**
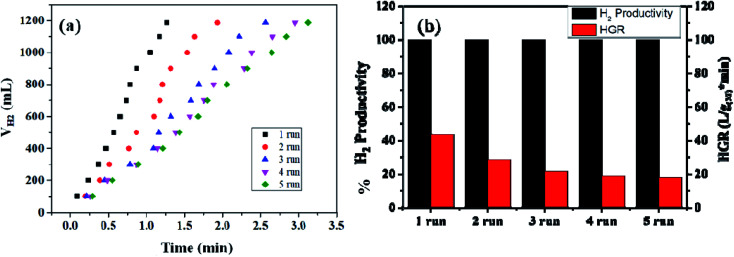
(a) Recyclability test of the Co_0.97_Pt_0.03_/CeO_*x*_/CGO nanocomposite for the ethanolysis of SB (13.21 mM) up to 05 runs at 300 K. (b) The hydrogen productivity and hydrogen generation rate (HGR) of Co_0.97_Pt_0.03_/CeO_*x*_/CGO during the reusability test.

The reusability of the nanohybrid was determined by consequently injecting 13.21 mmol of alkaline SB solution in the same reaction mixture for up to 09 cycles of ethanolysis. Consistent dehydrogenation performance was observed up to 07 cycles with a slightly decreased HGR. The reusability performance of the nanohybrid is represented in Fig. 8(b) and S13.† However, a drastic decline in the HGR was observed in the 08^th^ and 09^th^ cycles. This decline in the catalytic activity can be attributed to the excess accumulation of NaB(OC_2_H_5_)_4_ on the catalytic surface, which may deteriorate the active sites of the nanohybrid while also simultaneously poisoning the metallic platinum. Despite the accumulation of the reaction byproducts in the same reactor vessel, nearly 100% dehydrogenation activity has been obtained from the 1^st^ to 9^th^ cycle, which itself represents the potential of the nanohybrid for on-board hydrogen production. Simultaneously, the recyclability and %H_2_ productivity of a similar composite has been checked for five runs of ethanolysis of NaBH_4_ at similar experimental conditions [Fig. 9(a) and (b)]. After each run, the same catalyst is recovered, washed, and reused for the next run. The composite has shown complete dehydrogenation activity but the overall HGR is steadily decreased, which may due to the continuous deterioration of active sites.

### 3.3. Mechanistic elucidation for the ethanolysis of sodium borohydride

**Fig. 10:**
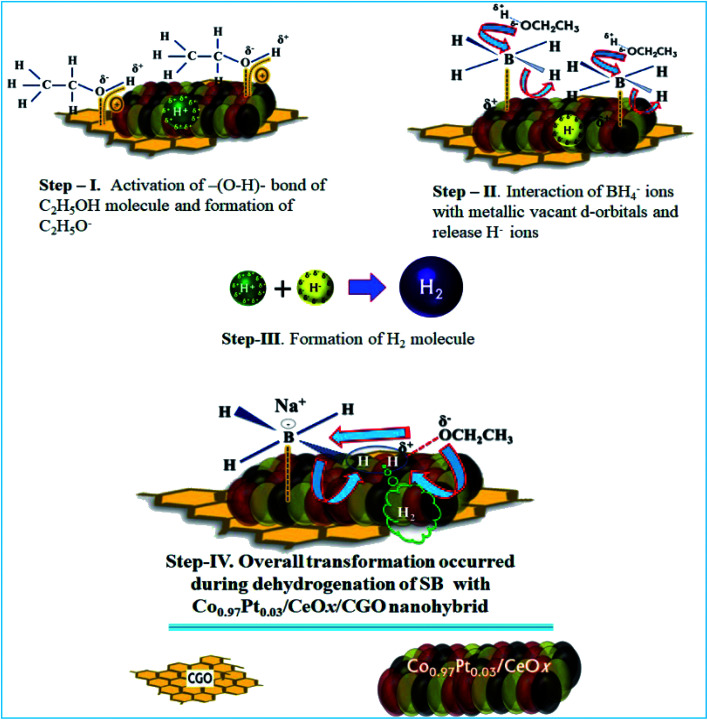
The proposed catalytic mechanism for the dehydrogenation of SB with the Co_0.97_Pt_0.03_/CeO_*x*_/CGO nanohybrid.

From the physico-chemical characterization of the nanohybrid and the experimental observations, it can be proposed that the evolved H_2_ molecule is formed by the combination of H^+^ ions from ethanol molecules and the H^−^ ions from the BH_4_^−^ molecule.^17,35,67^ During the ethanolysis of SB, the simultaneous formation of H^+^ and H^−^ ions is schematically shown in Fig. 10; each step involved in the dehydrogenation process is illustrated as follows.

The proposed mechanism for the catalytic ethanolysis of SB is summarized as follows:

(i) In the Co_0.97_Pt_0.03_/CeO_*x*_/CGO nanohybrid, the present mesoporous channels and surface oxygen functionalities on CGO provide an anchoring site for metal cations and simultaneously facilitate the adsorption of ethanol molecules by intermolecular H-bonding.

(ii) The adsorbed ethanol molecules can strongly interact with the available metal oxides, *i.e.*, CeO_*x*_ and Co_3_O_4_ by dipole–dipole interaction, as shown in step-I. The acidic hydrogen of ethanol is attracted towards the metallic O_2_^−^ ion while the vicinal oxygen atom (2p) of ethanol interacts with the Lewis cationic site, *i.e.*, Ce^4+^/Ce^3+^. This acid–base interaction can weaken the O–H bond of ethanol and facilitate the formation of H^*δ*+^ ions.

(iii) Simultaneously, BH_4_^−^ from NaBH_4_ can effectively bind with active platinum and cobalt nanoparticles by the transfer of charges from the electron-rich B atom to the vacant metallic d-orbital (Step-II). As a result, the strength of –(B–H)– becomes weak and the hydride ions are released, with a net positive charge on boron atom. This electron-deficient B atom further combines with ethoxide ions (OC_2_H_5_^−^) and forms ethoxy borate.

(iv) The hydride ion (H^−^) reacts with the proton obtained in Step-I and leads to the formation of the H_2_ molecule. (Step-III).H^+^ + H^−^ → H_2_

In the case of 100% dehydrogenation, the SB molecules can produce four moles of H_2_. The overall transformation of H_2_ formation on the surface of Co_0.97_Pt_0.03_/CeO*x*/CGO is presented in Step-IV.

The above proposed dehydrogenation pathway reveals that the synergetic interaction between the catalytic support CGO and ternary Co_0.97_Pt_0.03_/CeO_*x*_ can determine the rate and extent of ethanolysis. In the long term, the simultaneous generation and accumulation of ethoxy borate may hinder the catalytic activity by blocking the active metallic sites and the catalytic activity can be regenerated by the repeated washing of the nanocomposite.

## 4. Conclusion

We have demonstrated the applicability of the Co_0.97_Pt_0.03_/CeO_*x*_/CGO nanohybrid for the alkaline ethanolysis of SB, which exhibits 100% dehydrogenation activity at 300 K. The experimental investigations indicate that the catalytic support CGO and synergistic interaction among metallic Pt, Co/Co_3_O_4_, and CeO_*x*_ counterparts are typically responsible for the improved catalytic performance. In the Co_0.97_Pt_0.03_/CeO_*x*_/CGO nanohybrid, the available mesoporous channels on CGO and its inherent oxygen functionality particularly facilitate the distribution of metal nanoparticles. Moreover, the intercalated GO layers promote intermetallic charge transport. The characterization details suggest that the nanohybrid is composed of metal oxides, *i.e.*, Co_3_O_4_, CeO_*x*_ (Ce^4+^/Ce^3+^), and metallic Co and Pt NPs, which strongly interact with the vicinal C_2_H_5_OH molecule and BH_4_^−^ ions, respectively. On the basis of physicochemical characterization, we have proposed that hydrogen generation from SB is accompanied by a combination of H^−^ from BH_4_^−^ molecules and H^+^ from the C_2_H_5_OH molecule. The kinetic studies of SB ethanolysis suggested that the rate of hydrogen production is proportional to the cobalt concentration and is independent of the SB concentration. Overall, the Co_0.97_Pt_0.03_/CeO_*x*_/CGO catalyst has shown unprecedented hydrogen generation rate [41.53 L (min g_*M*_)^−1^], low activation energy [21.42 kJ mol^−1^], and excellent reusability [9 cycles], which makes it a highly competitive catalyst for the ethanolysis of SB. Therefore, we believe that this low-cost Co_0.97_Pt_0.03_/CeO_*x*_/CGO nanohybrid has great potential as a dehydrogenation catalyst and can be effectively used for on-board H_2_ production.

## Conflicts of interest

There are no conflicts to declare.

## Supplementary Material

RA-010-C9RA10742H-s001
