# Supplementary material for: Enhanced H2 production from dehydrogenation of sodium borohydride over the ternary Co0.97Pt0.03/CeOx nanocomposite grown on CGO catalytic support
Source: RSC Adv. 2020 Oct 16;10(63):38184–95. doi: 10.1039/c9ra10742h (PMC9119289; doi:10.1039/c9ra10742h)
Supplement: RA-010-C9RA10742H-s001 [file RA-010-C9RA10742H-s001.pdf]

Electronic Supplementary Material (ESI) for RSC Advances.
This journal is © The Royal Society of Chemistry 2020

Supplementary Information

Enhanced H_2 production from Ethanolysis of Sodium borohydride over ternary $Co_{0.97}Pt_{0.03}/CeO_x$ nanocomposite grown on catalytic support CGO

Abhay Vijay Kotkondawar[+] *and Sadhana Rayalu*[+*]

+Environmental Materials Division, CSIR-National Environmental Engineering Research Institute, Nehru Marg, Nagpur-440020, Maharashtra (M.S.), India.

***Corresponding Author:** s_rayalu@neeri.res.in

KEYWORDS: Ethanolysis; CGO; Sodium borohydride; Ammonia borane; hydrogen production; Activation Energy.

- Number of pages: 11
- Number of figures: 13
- Number of tables: 02

Content:

Figure.S1 FT- IR spectrum of carbon nanoparticles and CGO.

Figure.S2 Raman spectrum of carbon nanoparticles (CNP), CGO and $Co_{0.97}Pt_{0.03}$/CeOx/CGO nanohybrid.

Figure.S3 The Energy dispersive X-ray (EDX) spectrum of $Co_{0.97}Pt_{0.03}$/CeO*x*/CGO.

Figure.S4 The High resolution O1s XPS spectrum of CGO.

Figure.S5 XPS Survey spectrum of $Co_{0.97}Pt_{0.03}$/CeOx/CGO.

Figure.S6 Rate of H_2 generation (HGR) (L/$g_{(M)}$*min) from ethanolysis of SB catalyzed by nanohybrid with support ($Co_{0.97}Pt_{0.03}$/CeO*x*/CGO; Co/CeO*x*/CGO; $Co_{0.97}Pt_{0.03}$/CGO; CeOx/$Pt_{0.03}$/CGO; Co/CGO) and without support ($Co_{0.97}Pt_{0.03}$/CeO*x*; Co/CeO*x*; $Co_{0.97}Pt_{0.03}$; CeOx/$Pt_{0.03}$; Co)

Figure.S7 Effect of catalytic support (S: CNP, GO and CGO) on rate of hydrogen production from alkaline ethanolysis of SB catalyzed by $Co_{0.97}Pt_{0.03}$/CeO*x*/S nanohybrid.

Figure.S8 Effect of solvotic medium on rate of hydrogen production from alkaline ethanolysis of SB catalyzed by $Co_{0.97}Pt_{0.03}$/CeO*x*/CGO nanohybrid.

Figure.S9 Plot of the volume of H_2 generated versus time (min) for ethanolysis of SB (13.21 mM) catalyzed by $Co_{0.97}Pt_{0.03}$/CeO*x*/CGO at 300 K for different concentration of NaOH.

Figure.S10 Rate of H_2 generation (HGR) (L/g(M)*min) from ethanolysis of SB (13.21 mM) catalyzed by $Co_{0.97}Pt_{0.03}$/CeO*x*/CGO at 300 K for different concentration of NaOH

Figure.S11 Effect of Co-concentration on rate of hydrogen production from alkaline ethanolysis of SB catalyzed by $Co_{0.97}Pt_{0.03}$/CeO*x*/CGO nanohybrid.

Figure.S12 Effect of temperature on rate of hydrogen production from alkaline ethanolysis of SB catalyzed by $Co_{0.97}Pt_{0.03}$/CeO*x*/CGO nanohybrid.

Figure.S13 Reusability of $Co_{0.97}Pt_{0.03}$/CeO*x*/CGO nanohybrid for alkaline ethanolysis of SB.

Table.S1 Summary of deconvolution analysis of CGO.

Table.S2 Summary of deconvolution analysis of $Co_{0.97}Pt_{0.03}/CeO_x/CGO$.

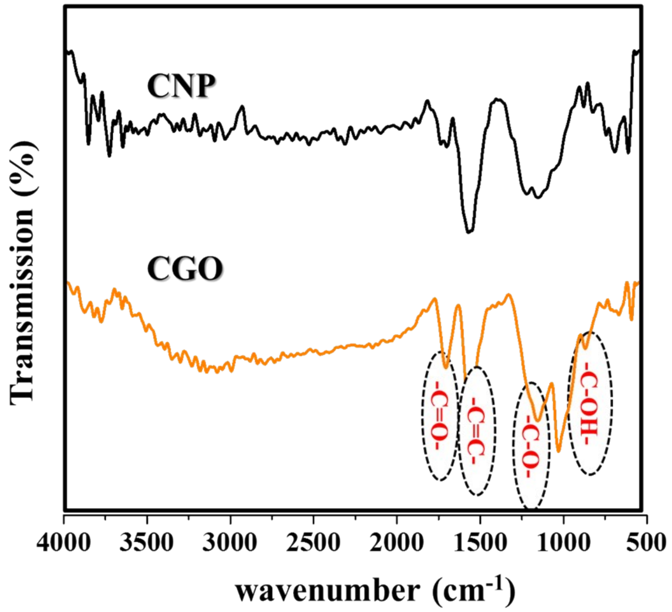

Figure. S1. FT-IR spectrum of carbon nanoparticles and CGO.

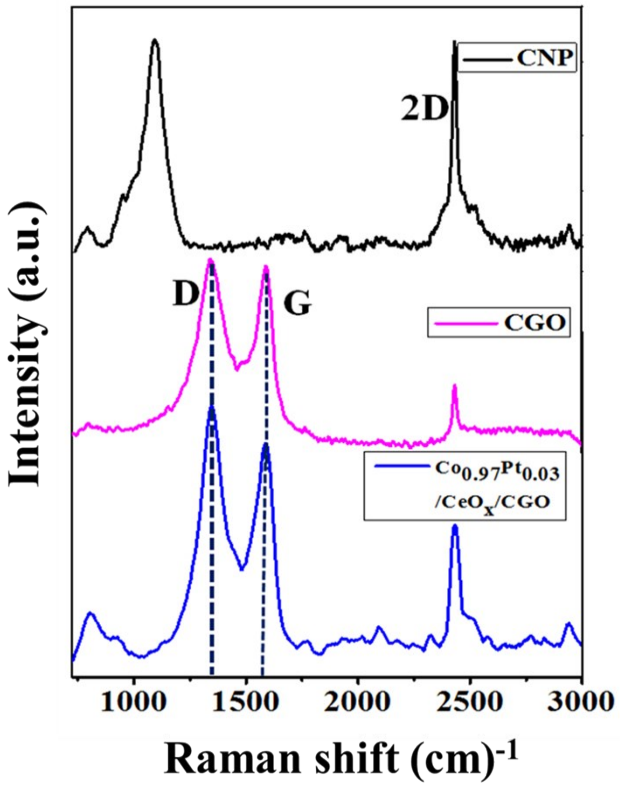

Figure. S2. Raman spectrum of carbon nanoparticles (CNP), CGO and $Co_{0.97}Pt_{0.03}$/CeOx/CGO nanohybrid.

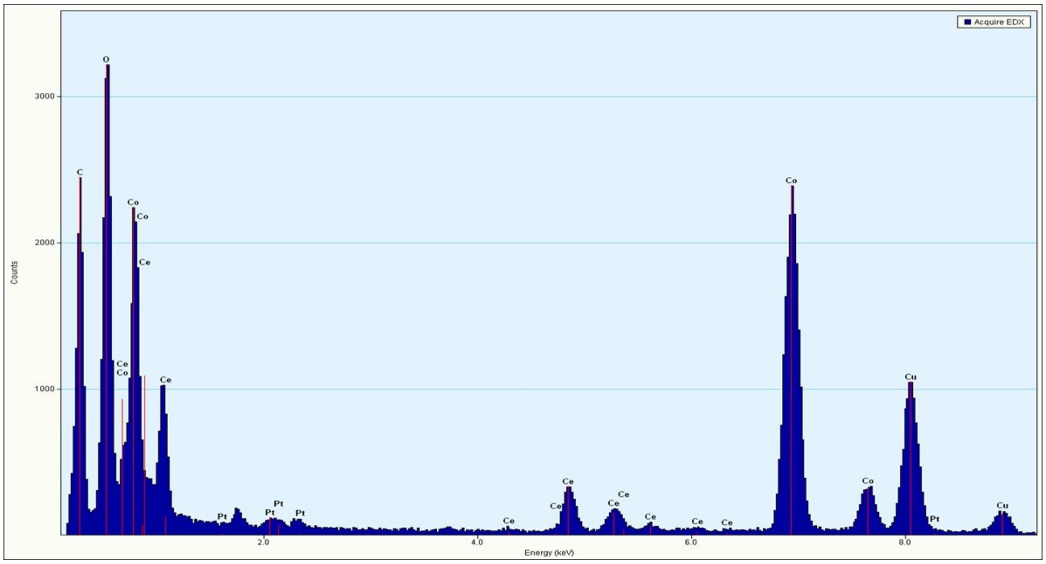

Figure. S3. The Energy dispersive X-ray (EDX) spectrum of $Co_{0.97}Pt_{0.03}/CeOx/CGO$.

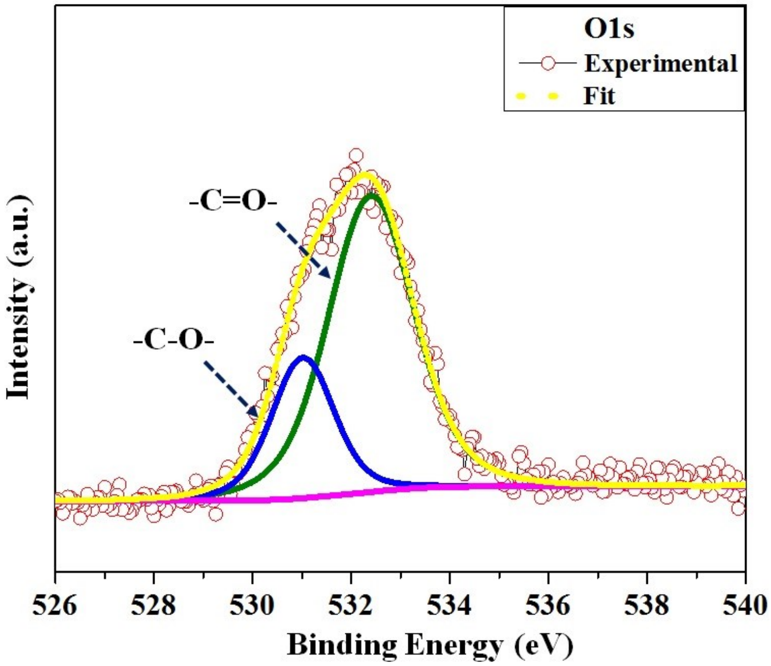

Figure. S4. The High resolution O1s XPS spectrum of CGO.

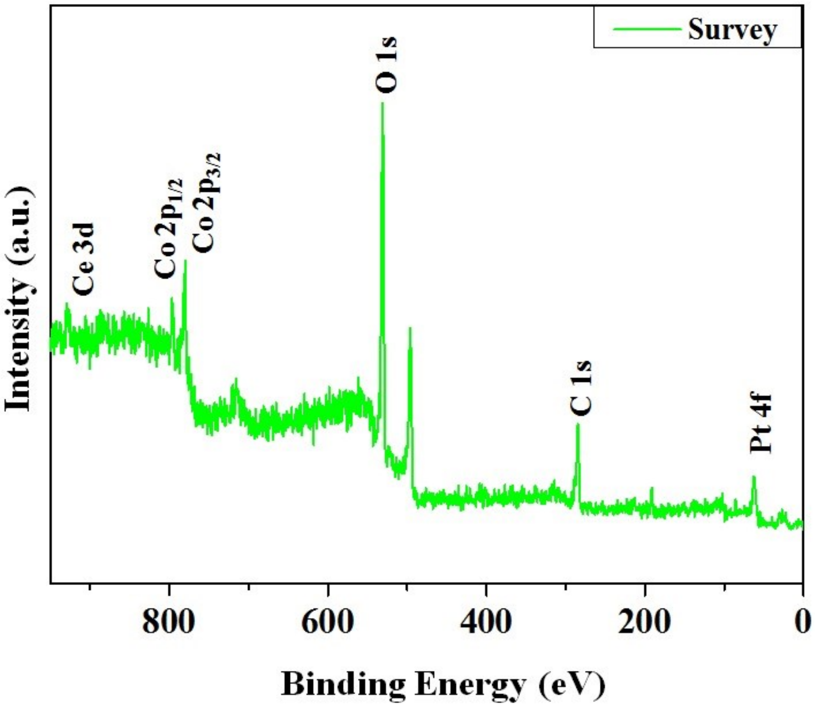

Figure. S5. XPS Survey spectrum of $Co_{0.97}Pt_{0.03}$/CeOx/CGO.

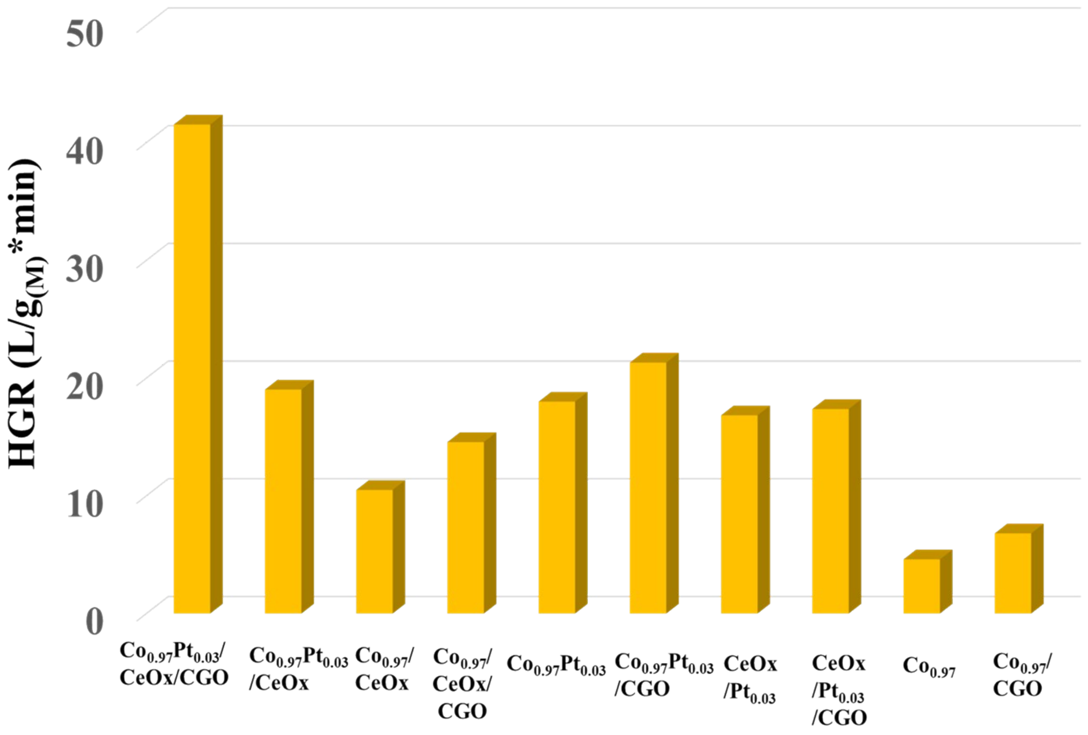

Figure. S6. Rate of H_2 generation (HGR) (L/$g_{(M)}$*min) from ethanolysis of SB catalyzed by nanohybrid with support ($Co_{0.97}Pt_{0.03}$/CeO*x*/CGO; Co/CeO*x*/CGO; $Co_{0.97}Pt_{0.03}$/CGO; CeOx/$Pt_{0.03}$/CGO; Co/CGO) and without support ($Co_{0.97}Pt_{0.03}$/CeO*x*; Co/CeO*x*; $Co_{0.97}Pt_{0.03}$; CeOx/$Pt_{0.03}$; Co).

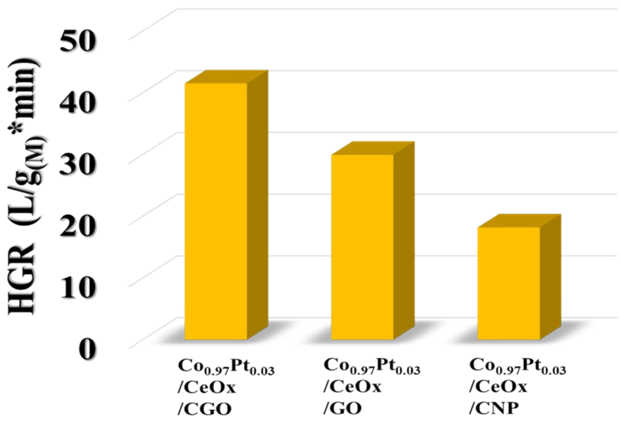

Figure. S7. Effect of catalytic support (S: CNP, GO and CGO) on rate of hydrogen production from alkaline ethanolysis of SB catalyzed by $Co_{0.97}Pt_{0.03}$/CeO*x*/S nanohybrid.

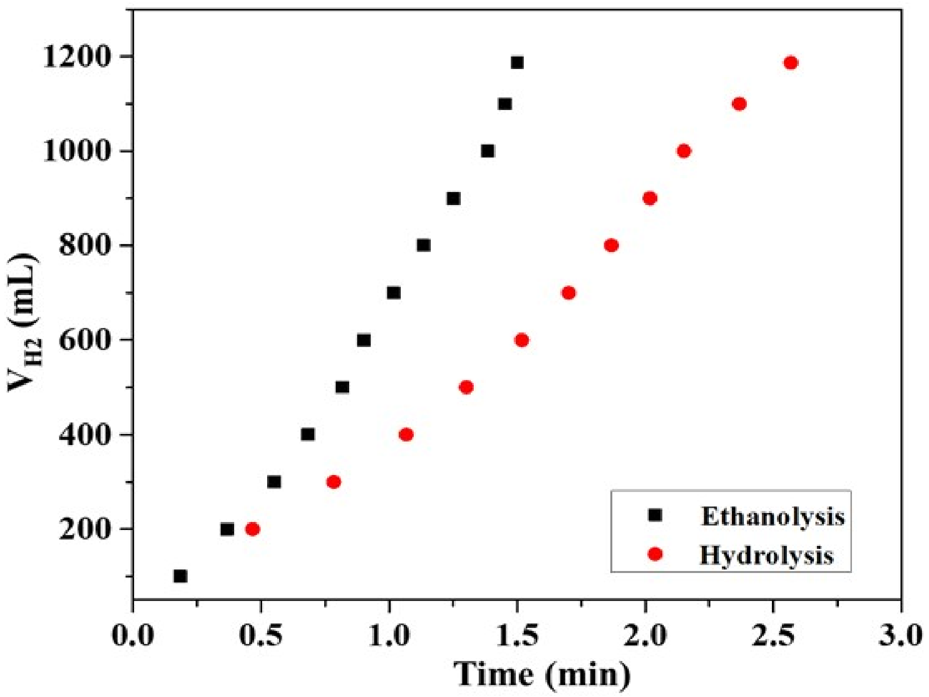

Figure. S8. Effect of solvotic medium on rate of hydrogen production from alkaline ethanolysis of SB catalyzed by $Co_{0.97}Pt_{0.03}/CeO\textit{x}/CGO$ nanohybrid.

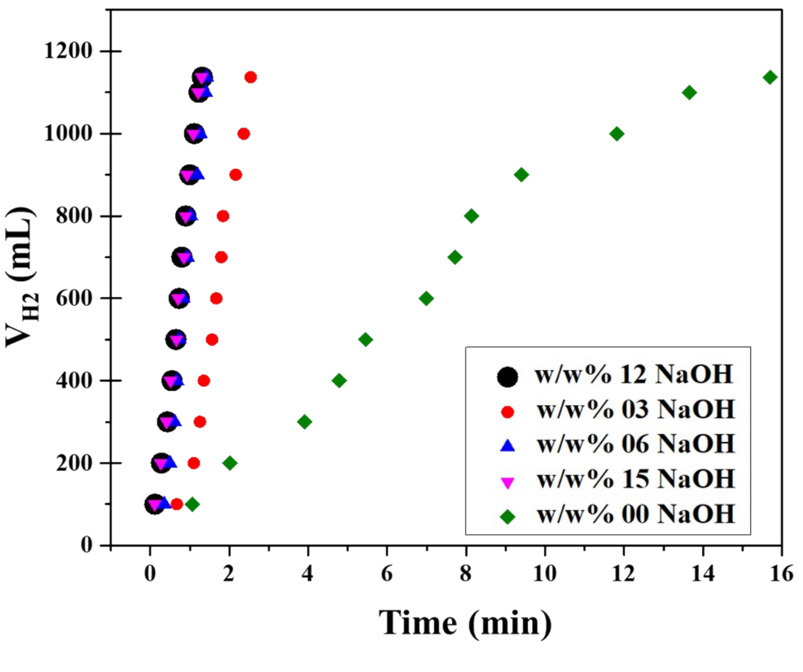

Figure.S9. Plot of the volume of H_2 generated versus time (min) for ethanolysis of SB (13.21 mM) catalyzed by $Co_{0.97}Pt_{0.03}/CeO\textit{x}/CGO$ at 300 K for different concentration of NaOH.

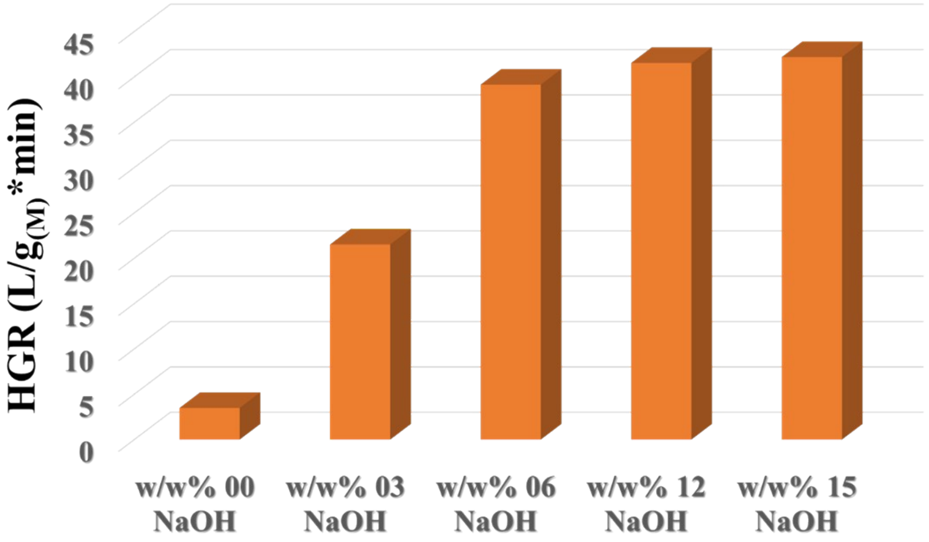

Figure.S10. Rate of H_2 generation (HGR) (L/g(M)*min) from ethanolysis of SB (13.21 mM) catalyzed by $Co_{0.97}Pt_{0.03}$/CeO*x*/CGO at 300 K for different concentration of NaOH.

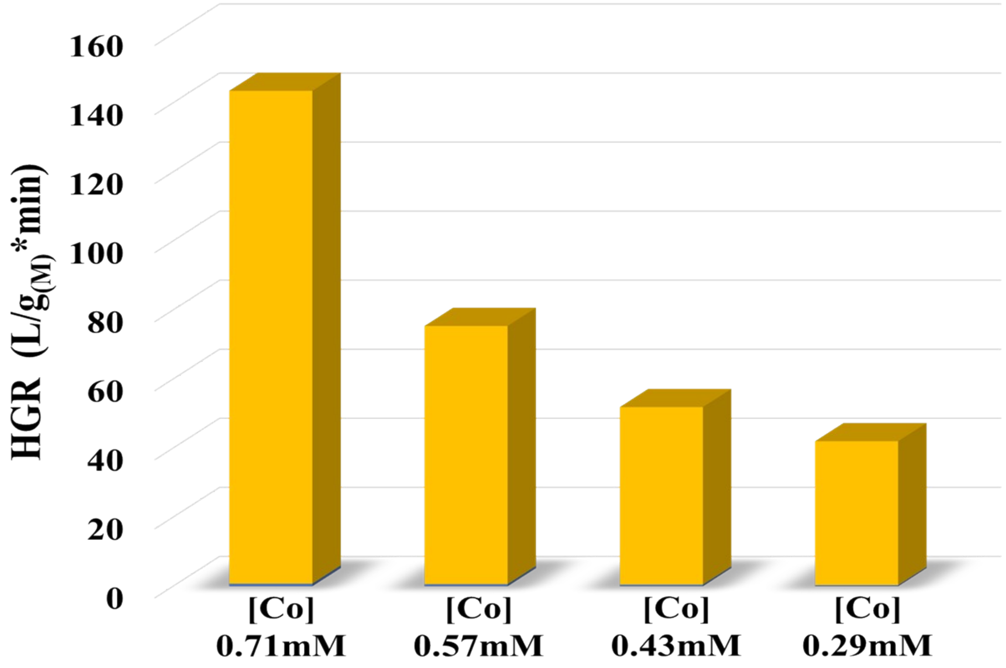

Figure. S11. Effect of Co-concentration on rate of hydrogen production from alkaline ethanolysis of SB catalyzed by $Co_{0.97}Pt_{0.03}$/CeO*x*/CGO nanohybrid.

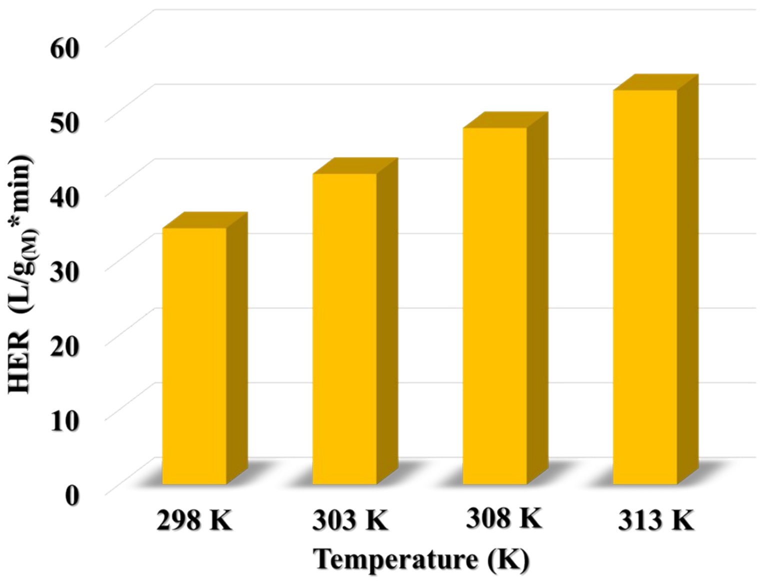

Figure. S12. Effect of temperature on rate of hydrogen production from alkaline ethanolysis of SB catalyzed by $Co_{0.97}Pt_{0.03}/CeO_x/CGO$ nanohybrid.

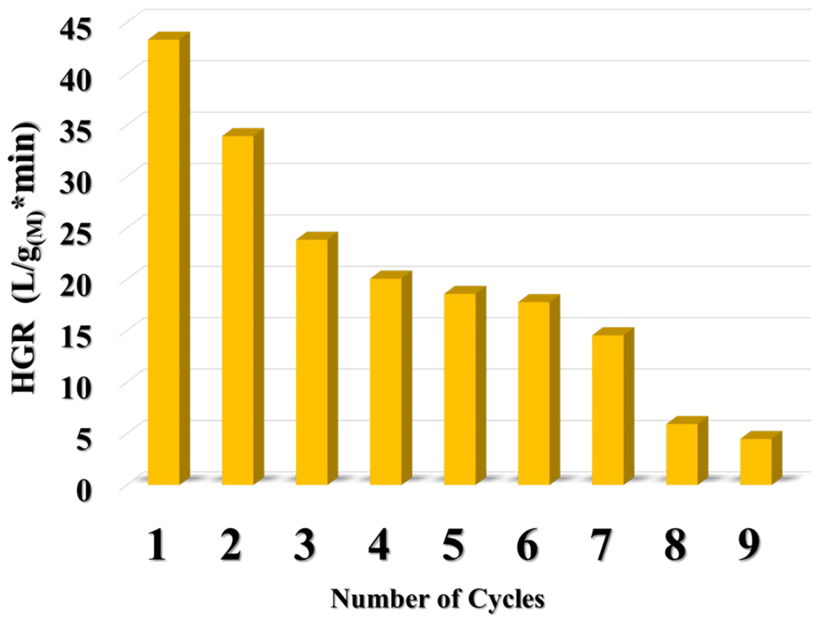

Figure. S13. Reusability of $Co_{0.97}Pt_{0.03}/CeO_x/CGO$ nanohybrid for alkaline ethanolysis of SB.

Table.S1: Summary of deconvolution analysis of CGO.

Catalyst	Functional Group	Binding Energy (eV)	% Total[a]
CGO	Csp2	283.96	55.9
	Csp3	285.4	11.8
	C-O	285.8	8.4
	O-C-O	286.4	10.2
	C=O	288.28	7.8
	O-C=O	289.06	5.9

a = % Total calculated by comparing peak area under the curve

Table. S2: Summary of deconvolution analysis of $Co_{0.97}Pt_{0.03}/CeO_x/CGO$.

Catalyst	Valance state	Binding energy (eV)	% Total[a]
Co-Pt-CeOx/CGO	Co (0)	778.08	25.5
	Co (II)	780.13	27.1
	Co (III)	795.2	47.4
	Ce (III)	903.48 (u')	21
		885.26 (v')	
	Ce (IV)	901.39 (u)	79
		907.09 (u")	
		916.93 (u"')	
		882.84 (v)	
		888.11 (v")	
		898.24 (v"')	
	Pt (0)	71.72<br>74.97	80.3
	Pt (II)	72.42<br>75.89	19.7

a = % Total calculated by comparing peak area under the curve
